# New Approach Using Ultrasonic Irrigant Activation and Propolis in Regenerative Endodontics: A Case Report

**DOI:** 10.1155/crid/2616239

**Published:** 2026-10-22

**Authors:** Luahra Peserico, Luís Eduardo Cechin, Camilla dos Santos Tibúrcio-Machado, Carlos Alexandre Souza Bier, Cláudia Medianeira Londero Pagliarin, Renata Dornelles Morgental

**Affiliations:** ^1^ Graduate Program in Dental Sciences Federal University of Santa Maria Santa Maria Rio Grande do Sul Brazil ufsm.br; ^2^ Endo APCD Institute São José dos Campos São Paulo Brazil

**Keywords:** calcium hydroxide, case report, propolis, regenerative endodontic procedure, ultrasonics

## Abstract

This study is aimed at describing a clinical case of a regenerative endodontic procedure (REP) in an immature permanent maxillary central incisor with pulp necrosis and apical periodontitis, using a modified protocol that included ultrasonic activation of 1.5% sodium hypochlorite (NaOCl) and 17% EDTA as irrigating solutions, along with an intracanal medicament (ICM) composed of propolis extract and calcium hydroxide (CH) powder, with a 7‐year follow‐up. An 8‐year‐old patient underwent an REP on Tooth 11 following trauma to the anterior teeth. Two weeks after procedure, a buccal sinus tract was observed. Cone‐beam computed tomography (CBCT) revealed a wide root canal, thin dentinal walls, a periapical osteolytic lesion, and an atypical apical formation resembling an “apical cap”, with no evidence of root development on the buccal aspect. With the aim of improving root canal disinfection, ultrasonic activation of the irrigating solutions was performed in cycles, first using 1.5% NaOCl, followed by 17% EDTA, and a final cycle with 1.5% NaOCl. A final rinse with saline was conducted before placement of the ICM, which consisted of propolis extract and CH powder. After 4 weeks, the patient remained asymptomatic, allowing a second REP to be performed. Seven years later, radiographic and tomographic examinations revealed continued root development, increased dentinal wall thickness, and new bone formation. This case report suggests that a second REP incorporating a modified disinfection strategy with ultrasonic activation of irrigating solutions and an ICM based on propolis and CH may be a feasible approach in selected immature permanent teeth with persistent infection following an initial regenerative procedure.

## 1. Introduction

Traditionally, apexification has been considered the treatment of choice for immature permanent teeth with pulp necrosis and apical periodontitis [1]. This procedure aims to induce the formation of a mineralized apical barrier or create an apical plug using biocompatible materials, thereby allowing root canal obturation [2]. However, because it does not promote continued root development, apexification results in the persistence of thin dentinal walls and an increased risk of root fracture [3]. These limitations have driven the development of regenerative endodontic procedures (REPs), which aim to promote continued root development and regeneration of the periapical tissues.

Although disinfection protocols for REPs have been proposed [4, 5], persistent intracanal infection remains one of the greatest challenges to treatment success. Failure rates may reach 40%, with approximately 14% of cases being attributed to persistent infection [6]. These findings indicate that establishing a biologically favorable environment requires not only effective microbial reduction but also preservation of the viability of the remaining tissues, highlighting the importance of optimizing disinfection strategies while preserving the biological conditions required for REPs.

Among the strategies proposed to enhance disinfection, irrigant activation has received considerable attention because of its ability to improve cleaning in anatomically complex areas of the root canal system. Although the risk of apical extrusion in immature teeth remains a concern [7], this event is not restricted to teeth with open apices and may also occur in teeth with complete root development [8, 9]. Furthermore, recent studies have shown that passive ultrasonic irrigation (PUI) enhances the release of dentin‐derived growth factors [10] without interfering with blood clot formation [11], suggesting that this approach may contribute to root canal disinfection while preserving the biological processes essential for regeneration.

Intracanal medicaments (ICMs) represent another critical component of this process. Given the need for agents that combine antimicrobial activity with high biocompatibility, propolis has emerged as a potential alternative to conventionally used ICMs. An experimental study demonstrated that propolis extract exhibited antibacterial activity comparable to that of traditional triple antibiotic paste (TAP), modified TAP, and calcium hydroxide (CH) against *Enterococcus faecalis [* *12* *]*. Furthermore, a randomized clinical trial in immature permanent teeth with necrotic pulps demonstrated that propolis achieved antimicrobial efficacy comparable to TAP when used as an ICM, supporting its potential use in REPs [13]. In addition, propolis has been associated with modulation of the inflammatory response, reduction of alveolar bone loss, and enhanced periodontal wound healing [14, 15]. Its high biocompatibility represents another important advantage, as previous studies have demonstrated its ability to preserve cell viability [16, 17].

Despite these advances, evidence regarding the management of persistent infection following an initial REP remains scarce. Therefore, the present case report describes the clinical management of persistent intracanal infection using a modified disinfection strategy incorporating PUI and propolis‐based ICM prior to performing a second REP.

## 2. Case Report

This case report was prepared in accordance with the CARE guidelines, and the completed CARE checklist is provided as Supporting Information (Available here). Assent was obtained from the minor participant, and written informed consent was obtained from the legal guardian prior to all clinical procedures.

An 8‐year‐old female patient was referred for evaluation 12 months after trauma to the maxillary anterior teeth, resulting in intrusion and crown fracture of Tooth 11. Following the injury, a conservative approach was adopted, consisting of restoration of the fractured coronal portion and clinical follow‐up, without repositioning, splinting, or antibiotic prescription. During the subsequent weeks, the tooth underwent spontaneous re‐eruption, returning to its original position. An initial periapical radiograph taken on the day of trauma at another dental clinic was provided by the patient′s guardian (Figure 1A). The radiograph revealed incomplete root development, corresponding to Nolla Stage 8 (approximately two‐thirds root formation), with a widely open apex.

**Figure 1:**
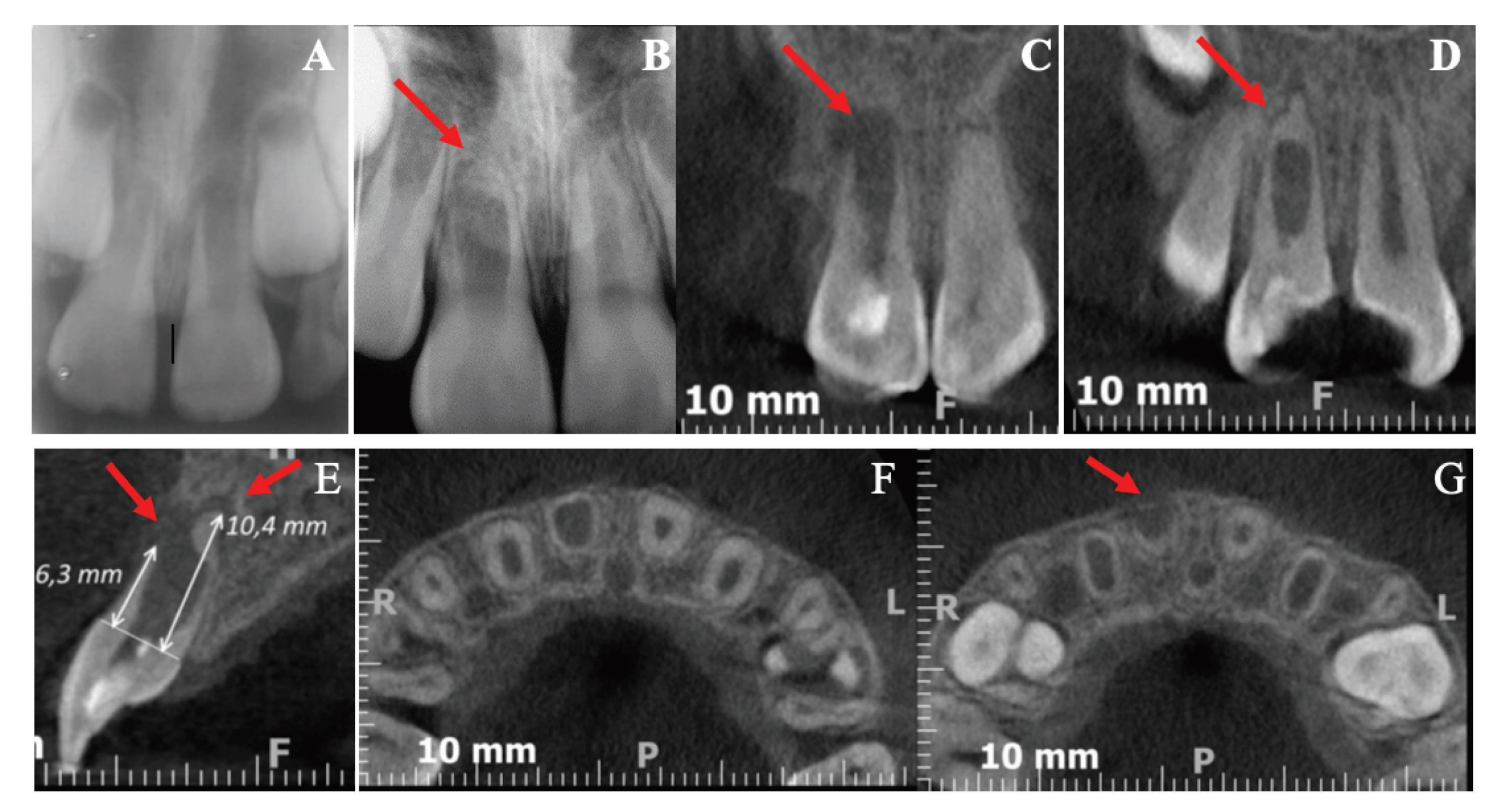
Periapical radiographs and CBCT images. (A) Periapical radiograph provided by the patient′s guardian, obtained on the day of trauma, showing incomplete root development of Teeth 11 and 21. (B) Preoperative radiograph obtained 12 months posttrauma. Continued root development of Tooth 21 is evident, approaching completion. Tooth 11 presents thin dentinal walls, a wide root canal, and an atypical apical formation (arrow). (C) Coronal CBCT view, showing an immature root with a wide canal and thin dentinal walls (arrow). (D) Coronal view showing an atypical apical formation (arrow). (E) Sagittal view revealing a periapical osteolytic lesion (arrow), absence of root formation on the buccal aspect of the apical third (arrow), periodontal ligament alterations, and bone loss. The palatal portion of the apical third of the root appears preserved. (F,G) Axial views demonstrating a wide root canal and resorption of the alveolar cortical bone in the buccal region (arrow).

The patient′s medical and dental history revealed no significant findings other than the dental trauma. Clinical examination revealed a minor crown fracture and a buccal sinus tract associated with Tooth 11. The tooth showed negative responses to percussion, apical palpation, and pulp sensitivity tests, and periodontal probing depths were within normal limits. The periapical radiograph (Figure 1B) showed normal root development of Tooth 21, whereas Tooth 11 presented incomplete and atypical root formation associated with a periapical lesion.

Considering the presence of pulp necrosis associated with incomplete root development, a widely open apex, and thin dentinal walls, and after discussing the available treatment options with the patient′s legal guardian, REP was selected because of its potential to promote continued root development, including increased root length, thickening of the dentinal walls, and apical closure. The procedure was performed in accordance with the American Association of Endodontists (AAE) clinical considerations for a regenerative procedure [4], with case‐specific adaptations.

### 2.1. Initial Regenerative Endodontic Protocol

After local anesthesia and rubber dam isolation, endodontic access was established. Drainage of purulent exudate was observed upon access to the pulp chamber. Root canal disinfection was performed with slow, copious irrigation using 1.5% sodium hypochlorite (NaOCl) (Nova Derme Compounding Pharmacy, Santa Maria, Rio Grande do S, Brazil). The working length (WL) was determined radiographically. Initially, a provisional tooth length was established based on the initial periapical radiograph. A size 80 K‐file was then inserted into the root canal to the estimated length, and a subsequent periapical radiograph was obtained to adjust and confirm the WL before instrumentation. Minimal mechanical instrumentation was performed to avoid further weakening of the thin dentinal walls. A size 80 K‐file was used in light contact with the canal walls solely to disrupt the intracanal biofilm, without intentional enlargement of the root canal. Due to persistent moisture within the root canal, an ICM was placed. A 1% propolis paste was formulated in a glycerin‐based vehicle (Nova Derme Compounding Pharmacy, Santa Maria, Rio Grande do S, Brazil) based on the method described by Pagliarini et al. [18]. CH powder was gradually added to the propolis paste until a homogeneous, toothpaste‐like consistency was achieved, consistent with a consistency‐based preparation previously described for endodontic pastes containing propolis and CH [19]. No standardized ratio of CH powder to propolis extract was used, and the exact amounts of the components were not recorded. The medicament was then delivered into the root canal using a 29‐gage NaviTip irrigation needle (Ultradent Products Inc., South Jordan, Utah, United States) and syringe.

Four weeks later, the patient was asymptomatic, and the REP was performed. The canal was irrigated with 20 mL of 17% ethylenediaminetetraacetic acid (EDTA), followed by 5 mL of sterile saline. A precurved size 60 K‐file was then introduced 2 mm beyond the apical foramen using rotational movements to induce bleeding into the canal. Approximately 15 min were allowed for the blood clot to form and stabilize. Bleeding was then controlled 2 mm below the cementoenamel junction (CEJ), and a resorbable matrix (Lumina Set, Criteria Biomaterials, São Carlos, São Paulo, Brazil) was positioned over the clot. For coronal sealing, mineral trioxide aggregate (MTA) (MTA Angelus, Londrina, Paraná, Brazil) was placed in the cervical third, and the tooth was restored with composite resin.

### 2.2. Modified Regenerative Endodontic Protocol Following Persisten Infection after the Initial Procedure

Two weeks after completion of the initial regenerative protocol, a persistent buccal sinus tract was observed, consistent with persistent infection. Cone‐beam computed tomography (CBCT), obtained at a private oral and maxillofacial radiology clinic using a limited field of view (FOV) focused on Tooth 11, revealed a wide root canal with extremely thin dentinal walls (Figure 1C) and a periapical osteolytic lesion (Figure 1D). An atypical apical formation resembling an apical cap was observed, with no evidence of root development on the buccal aspect (Figure 1E), whereas the palatal portion of the apical third remained intact (Figure 1F,G).

Considering the patient′s young age, incomplete root development, and extremely thin dentinal walls, a second REP was preferred over apexification. The persistence of the sinus tract was interpreted as a clinical sign of persistent intracanal infection, supporting modification of the disinfection strategy before a second REP. Therefore, the disinfection protocol was modified with the aim of improving microbial control before performing a second REP.

This time, PUI was performed using an E1 Irrissonic ultrasonic tip (ISO size 20, 0.01 taper; Helse Ultrasonic, Santa Rosa de Viterbo, São Paulo, Brazil) coupled to a Newtron Booster ultrasonic unit (Acteon, Mérignac, France) operated at 20% power, according to the manufacturer′s instructions [18]. The operating frequency (minimum output frequency: 28 kHz) was automatically controlled by the ultrasonic unit rather than manually set by the operator.

The ultrasonic tip was positioned 2 mm short of the WL. The irrigation protocol consisted of sequential activation of 5 mL of 1.5% NaOCl, followed by 5 mL of 17% EDTA, and a final activation with 5 mL of 1.5% NaOCl. Each irrigant was activated for three 20‐s cycles (total activation time of 1 min per irrigant), and the irrigating solution was refreshed after each activation cycle. A final irrigation was performed with sterile saline, the canal was dried with sterile paper points, and the previously described ICM was placed again.

Four weeks later, the patient remained asymptomatic. A second REP was then performed following the regenerative protocol previously described, including induction of intracanal bleeding, placement of a resorbable matrix, an MTA coronal barrier, and composite resin restoration.

### 2.3. Follow‐Up Examinations

During follow‐up, the patient remained asymptomatic and showed a positive response to pulp sensitivity testing. Radiographs obtained at 3 and 24 months (Figures 2A,B) demonstrated progressive root elongation and dentinal wall thickening. Four years after the regenerative treatment, fixed orthodontic treatment using the advanced MBT system (Morelli, Sorocaba, São Paulo, Brazil) was initiated to correct Class II malocclusion involving the molars and canines. At the 7‐year follow‐up, radiographic and CBCT examinations revealed healing of the periapical tissues (Figure 2C) and evidence of new bone formation in the buccal region, despite the persistence of a small contour defect in the buccal aspect of the apical third of the root (Figures 2D,E).

**Figure 2:**
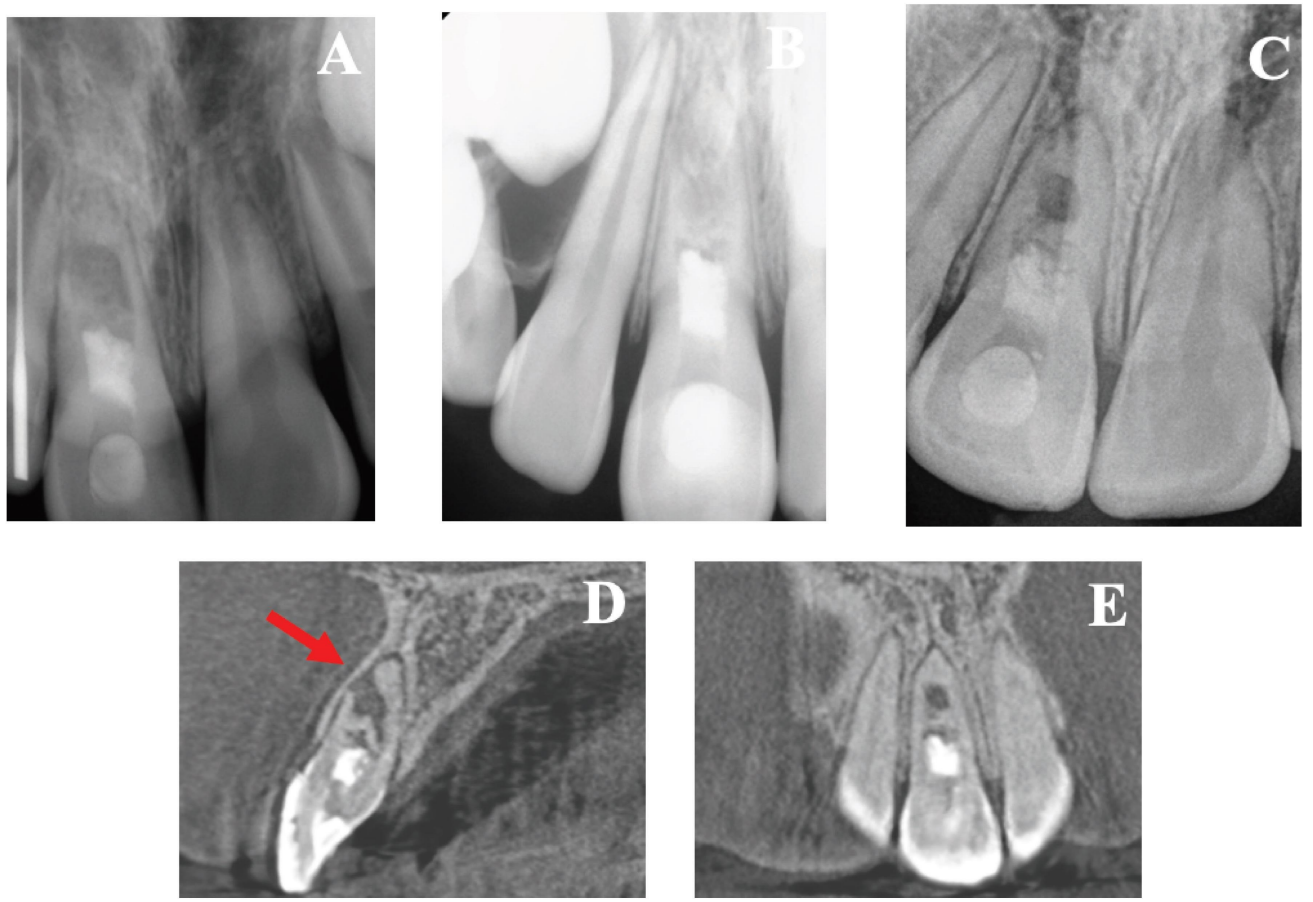
Postoperative periapical radiographs and CBCT images. (A,B) Periapical radiograph obtained at 3 and 24 months after REP, respectively, progressive root elongation and dentinal wall thickening. (C) At the 7‐year follow‐up, the periapical radiograph shows healing of the periapical tissues. (D,E) Sagittal and coronal CBCT views, respectively, showing absence of the previously observed periapical osteolytic lesions and evidence of new bone formation in the buccal region. A small residual contour defect is visible on the buccal aspect of the apical third (arrow).

## 3. Discussion

The success rate of REPs varies widely, ranging from 60% to 90%. As with conventional endodontic treatment, the predictability of REPs is closely linked to the effectiveness of root canal disinfection; however, this factor alone is insufficient, as outcomes depend on the interplay of multiple biological and clinical variables. In this case, the combination of therapeutic strategies, including irrigant activation and ICM use, was intended to improve root canal disinfection and may have contributed to the favorable clinical outcome.

Mechanical instrumentation is limited in its ability to remove biofilms because of anatomical complexities, making irrigating solutions indispensable [20]. This limitation becomes particularly relevant in immature teeth, in which the fragility of dentinal walls contraindicates aggressive instrumentation [5]. For this reason, current guidelines recommend specific irrigation protocols for REPs. The AAE recommends the use of 1.5% NaOCl, whereas the European Society of Endodontology (ESE) suggests concentrations ranging from 1.5% to 3% [4, 5]. Higher concentrations may compromise the viability and differentiation potential of stem cells from the apical papilla; therefore, lower concentrations are preferred [21]. In addition, both organizations recommend the use of 17% EDTA prior to induction of intracanal bleeding because of its role in growth factor release and dentin conditioning [22].

Ultrasonic activation of the irrigating solutions was employed because of persistent clinical signs of infection in a tooth with incomplete and atypical root morphology. Such anatomical complexity may favor biofilm retention and hinder the effectiveness of conventional irrigation. However, the AAE and ESE guidelines do not provide specific recommendations for irrigant activation in REPs [4, 5], possibly because of concerns related to apical extrusion and its impact on cell viability. The literature on REPs is heterogeneous [23], particularly in teeth with open apices, and optimization of disinfection protocols remains under investigation. In vitro studies have demonstrated that different activation techniques may result in irrigant and debris extrusion; however, these findings should be extrapolated to clinical practice with caution, given the limitations of these models in adequately simulating periapical tissues [7, 8]. Beyond the well‐established benefits of irrigant activation, recent evidence suggests that EDTA agitation may increase the release of dentin‐derived growth factors essential for cellular differentiation, without compromising the stability of the blood clot, a key element for REP success [10].

Although REPs may be performed in a single visit [24], the use of an ICM with antibacterial activity may be indicated to support root canal disinfection. According to the AAE, either CH or low‐concentration triple TAP may be used as an ICM [4], whereas the ESE recommends CH as the preferred option [5]. In the present case, CH powder combined with propolis paste was selected based on its favorable biological properties.

The selection of propolis as a component of the ICM was based on experimental evidence demonstrating antimicrobial activity against microorganisms associated with endodontic infections. In dentinal tubule infection models using *E. faecalis*, propolis exhibited antimicrobial activity comparable to or greater than that of CH [25, 26]. Furthermore, when incorporated into CH pastes, propolis maintained antimicrobial activity against *E. faecalis* and multispecies biofilms, particularly during the first 7 days of exposure [27]. Collectively, these findings support the biological plausibility of propolis as an adjunctive component of an ICM in regenerative protocols in which infection control and preservation of biologically favorable environment are important treatment considerations.

In addition to its antimicrobial activity, the rationale for selecting propolis was also based on its favorable biological profile. Compared with TAP, propolis exhibits lower cytotoxicity, which may help preserve an intracanal environment more conducive to cell survival [18, 25]. This rationale is further supported by El‐Tayeb et al. [28], who demonstrated in an experimental model involving immature permanent teeth with pulp necrosis in dogs that propolis paste achieved antibacterial efficacy and regenerative outcomes comparable to those of TAP, including increased root length, dentinal wall thickening, apical closure, and vital tissue formation.

Although derived from different experimental and clinical models, the available evidence suggests that propolis possesses antimicrobial, anti‐inflammatory, and immunomodulatory properties that may be relevant to REPs. A clinical study demonstrated that propolis, when used as an ICM in immature permanent teeth, exhibited antimicrobial efficacy comparable to that of TAP, supporting its potential as a biocompatible alternative for use in REPs [13]. More recently, a systematic review of randomized clinical trials concluded that propolis is a promising adjunct in endodontic treatment because of its antimicrobial, anti‐inflammatory, and wound‐healing properties, while emphasizing the need for further high‐quality clinical studies [29]. In addition, Da Silva et al. [30] demonstrated that systemic administration of green and red propolis reduced inflammation and bone resorption in an experimental model of apical periodontitis, further supporting the biological plausibility of a potential role for its bioactive compounds in periapical tissue repair.

Despite these promising findings, evidence regarding the combination of CH and propolis remains limited. In an experimental model of delayed tooth replantation, the addition of 10% propolis, with or without ultrasonic activation, did not result in significant histological improvement compared with CH alone, suggesting that the addition of propolis to CH does not necessarily result in improved biological outcomes and that any potential clinical advantage over CH alone to be established [31].

From a physicochemical perspective, the incorporation of propolis also appears not to compromise the alkalizing properties of CH. Montero and Mori [32] demonstrated that a CH–propolis paste promoted the diffusion of hydroxyl ions through dentin, as evidenced by the progressive increase in the pH of the surrounding medium, particularly after 72 h. These findings suggest that the addition of propolis may not impair the alkalizing ability of CH, a property associated with its antimicrobial activity; although there is still insufficient evidence to conclude that this combination provides additional clinical benefits.

This case report describes favorable clinical and radiographic outcomes following a second REP in a tooth with atypical apical root development and a defect in the buccal root wall. The atypical root morphology may be related to the history of intrusive luxation, which may have interfered with normal root development. The persistence of the buccal defect in the apical third was observed after subsequent orthodontic treatment; however, whether orthodontic forces contributed to this finding cannot be determined. The available evidence remains inconclusive regarding the impact of orthodontic tooth movement on teeth undergoing REPs after traumatic injury [5, 33].

The main limitations of this case report include the inability to attribute the favorable clinical outcome to a single intervention, as the modified regenerative protocol involved multiple simultaneous modifications, including PUI, an irrigation sequence using 1.5% NaOCl, 17% EDTA, and an ICM composed of CH and propolis. Therefore, it is not possible to determine the individual contribution of each component to the clinical and radiographic outcomes observed. In addition, the nature of the tissue formed within the root canal could not be confirmed histologically, which is an inherent limitation of clinical reports on REPs in humans. Despite these limitations, the 7‐year clinical and radiographic follow‐up represents a major strength of this case, providing a substantially longer observation period than is typically reported in the literature and offering valuable information regarding the long‐term stability of the treatment outcomes [34].

In conclusion, this case report suggests that a second REP may be associated with favorable clinical and radiographic outcomes following persistent infection after the initial REP. In this case, retreatment incorporated additional strategies aimed at improving root canal disinfection, including PUI and an ICM composed of CH and propolis. However, because multiple interventions were modified simultaneously and microbial reduction was not directly assessed, the contribution of each individual strategy to the favorable outcome cannot be determined. Furthermore, this case expands the available evidence by documenting favorable long‐term outcomes in a tooth with atypical apical root development and a buccal root wall defect, over a 7‐year clinical and radiographic follow‐up. These findings contribute to the limited body of evidence regarding the long‐term outcomes of modified regenerative endodontic protocols in anatomically complex cases.

## Author Contributions

Conceptualization: Luahra Peserico, Camilla dos Santos Tibúrcio‐Machado. Data curation: Luahra Peserico, Camilla dos Santos Tibúrcio‐Machado. Investigation: Camilla dos Santos Tibúrcio‐Machado, Carlos Alexandre Souza Bier, Cláudia Medianeira Londero Pagliarin. Methodology: Camilla dos Santos Tibúrcio‐Machado, Cláudia Medianeira Londero Pagliarin. Project administration: Luahra Peserico. Supervision: Luahra Peserico, Camilla dos Santos Tibúrcio‐Machado, Renata Dornelles Morgental. Visualization: Luahra Peserico. Writing—original draft: Luahra Peserico, Luís Eduardo Cechin. Writing—review and editing: Luahra Peserico, Camilla dos Santos Tibúrcio‐Machado, Renata Dornelles Morgental.

## Funding

This study was supported by the Conselho Nacional de Desenvolvimento Científico e Tecnológico, 10.13039/501100003593, 168287/2023‐4.

## Disclosure

All authors have read and approved the final version of the manuscript. The corresponding author, Luahra Peserico, had full access to all the data in this study and takes full responsibility for the integrity of the data and the accuracy of the data analysis.

## Ethics Statement

The study was reviewed and approved by the local ethics committee under protocol number 85474924.6.0000.5346.

## Conflicts of Interest

The authors declare no conflicts of interest.

## Supporting information


**Supporting information** Additional supporting information can be found online in the Supporting Information section. Data S1: CARE checklist of information to include when writing a case report.

## Data Availability

The data that support the findings of this study are available upon request from the corresponding author.
